# General Microstructure Factor Analysis of Diffusion MRI in Gray-Matter Predicts Cognitive Scores

**Published:** 2025-10-28

**Authors:** Lucas Z. Brito, Ryan P. Cabeen, David H. Laidlaw

**Affiliations:** a Department of Physics, Harvard University, Cambridge, MA, 02140, USA; b Department of Physics, Brown University, Providence, RI, 02912, USA; c Laboratory of Neuro Imaging, USC Mark and Mary Stevens Neuroimaging and Informatics Institute, Keck School of Medicine of USC, University of Southern California, Los Angeles, CA, 90033, USA; d Department of Computer Science, Brown University, Providence, RI, 02912, USA

**Keywords:** NODDI, diffusion MRI, microstructure, general factor, cognition, gray matter

## Abstract

Diffusion MRI has revealed important insights into white matter microstructure, but its application to gray matter remains comparatively less explored. Here, we investigate whether global patterns of gray-matter microstructure can be captured through neurite orientation dispersion and density imaging (NODDI) and whether such patterns are predictive of cognitive performance. Our findings demonstrate that PCA-based global indicators of gray-matter microstructure provide complementary markers of structure–function relationships, extending beyond region-specific analyses. Our results suggest that general microstructure factors may serve as robust, interpretable biomarkers for studying cognition and cortical organization at the population level. Using diffusion MRI and behavioral data from the Human Connectome Project Young Adult study, we derived region-averaged NODDI parameters and applied principal component analysis (PCA) to construct general gray-matter microstructure factors. We found that the factor derived from isotropic volume fraction explained substantial inter-individual variability and was significantly correlated with specific cognitive scores collected from the NIH Toolbox. In particular, the isotropic volume fraction factor was linked to reading and vocabulary performance and to cognitive fluidity.

## 1. Introduction

Diffusion MRI study of gray-matter microstructure is an active field of research, comparatively less developed than diffusion MRI techniques aimed at white matter regions [1]. Diffusion imaging of gray matter is subtler for a number of reasons—for instance, the geometry of the cortical sheets makes voxels more sensitive to CSF partial volume effects, and the complex and comparatively isotropic tissue microstructure complicates detection of cellular architecture via diffusion signal [1, 2]. Nonetheless, success has been achieved in revealing gray-matter microstructure with diffusion scans, for instance at the structural level via sensitivity to laminar or regional microstructural heterogeneity [3, 4] or anisotropy in developing cortices [5]. Thus, much inquiry has been directed at revealing correlations between microstructure and function via diffusion imaging, with a particular emphasis on cognition [6, 7, 8, 9, 10].

One way to extract more specific information from diffusion signals is to fit the scan to a multicompartment model. Although diffusion MRI acquisitions can provide, on their own, much anatomically relevant information to clinicians and researchers, often richer information is revealed by fitting acquisitions to biophysical models of tissue microstructure. In recent years, attention has been devoted to developing models capable of capturing the subtleties of gray matter specifically [11], but more generic models are also believed to reliably capture microstructural features. In this study, we employ one such model, neurite orientation dispersion and density imaging (NODDI), to study gray-matter microstructure [12]. NODDI is composed of an intracellular compartment that models diffusion in the region bounded by neurites, an extracellular compartment representing the vicinity of neurites, where flow is restricted by, for example, glial cells or somas, and an isotropic compartment corresponding to CSF. This is in contrast with more common imaging techniques such as diffusion tensor imaging (DTI), which is a single-compartment model consisting of a diffusivity tensor from which scalar measures such as fractional anisotropy (FA) are derived [13, 14, 15]. In this sense, it is best thought of as a signal representation as opposed to a multicompartment biophysical model [16].

The use of NODDI in structural studies is promising, as it demonstrates greater specificity than single-component DTI images [17, 18]. For example, NODDI has been shown to capture cognitive functional trends better than single-tensor approaches [7]. Consequently, it has become a common modality in studies of function–structure relationships centered on cognition and gray-matter microstructure. These include investigations of age-related changes in diffusivity between the hippocampal compartments [7], associations between intracellular volume fraction, $f_{\text{icvf}}$, and cognitive deficits in patients with thalamic stroke [19], and links between neurite density and Alzheimer’s disease [20]. However, relatively few studies have examined global associations between NODDI-derived microstructural measures and cognitive performance. Certain tasks are likely to involve widespread networks, with population-level variation reflecting coordinated changes across multiple regions. Such analyses have been conducted in white matter tracts, where a general factor of tract integrity—computed using principal component analysis (PCA)—was found to correlate significantly with an analogous general factor of processing speed [21]. In that case, approximately eight tracts contributed to a global indicator of white matter integrity. In this work, we build on that approach by computing a general factor across gray-matter regions and investigating its associations with cognitive performance. We call this measure the gray-matter general microstructure factor (GMF). Gray-matter introduces additional challenges and considerations: we employ NODDI as it is the simplest multi-compartment diffusion model capable of revealing relevant microstructural features, in contrast to the DTI-based approach used in the prior white matter study. Furthermore, the heightened sensitivity of gray matter to partial volume effects motivates us to introduce a weighted-average volume covariate to control for these confounds. Our analysis is conducted on a large cohort of approximately one thousand participants from the Human Connectome Project Young Adult (HCP-YA) [22].

Our paper is structured as follows: in Sec. 2.1 we review the acquisition protocol and preprocessing pipeline used in the HCP-YA, including registration of fitted NODDI parameter maps to Freesurfer cortical atlases. We also summarize the cognitive behavioral scores relevant to our study. In Sec. 2.3 we introduce the PCA-based analysis we perform on the NODDI maps, including our strategy for controlling for partial voluming. We find that the general microstructure factor generated by the first PCA component correlates significantly with reading and vocabulary and card sorting scores for $f_{\text{iso}}$. In Sec. 3 we discuss these results, and perform a series of posthoc tests to study the nature of these trends across regions and between regions. Lastly, we review our findings in Sec. 4.

## 2. Methods

### 2.1. DWI data

Data used was sourced from the Human Connectome Project Young Adult study (HCP-YA) 1200 subject release diffusion- and T1-weighted MRI acquisitions [23]. Acquisition was performed with the HCP protocol described in [22, 24], which we review here. Diffusion-weighted MRI data were collected with the Connectome Skyra Siemens 3-Tesla scanner, using a 32-channel head coil. T1-weighted images were acquired with the 3D MPRAGE sequence with 0.7mm isotropic resolution (FOV = 224mm, in-plane matrix size 320, and 256 slices in a single slab), with repetition time $T_{R}=2400$ ms, echo time $T_{E}=2.14$ ms, inversion time $T_{I}=1000$ ms, flip angle = 8°, bandwidth = 210Hz per pixel, echo spacing = 7.6ms, and phase encoding undersampling factor GRAPPA = 2.10%. Diffusion-weighted MRI images were acquired with a spin-echo EPI sequence with 1.25mm isotropic resolution (FOV RO×PE = 210×180, matrix RO×PE = 168×144) with 111 slices of thickness 1.25mm and a multiband factor of 3 with flip angles 68° and 160°. Each phase-encoding direction utilized single-diffusion left-to-right and right-to-left (L/R, R/R) encoding with *b* = 1000, 2000, and 3000smm^−2^, sampled with 18 baseline scans and 270 diffusion-weighted scans with $T_{E}=89$ ms and $T_{R}=5520$ ms. Each shell was acquired with 90 diffusion weighting directions, as well as an additional 6 b=0 acquisitions; these acquisitions are repeated twice for each encoding polarity. Diffusion-weighted MRI images were preprocessed with the HCP pipeline detailed in [25]. Relevant for our application is the usage of the L/R, R/L double phase encoding polarity to reduce motion, eddy-current, and susceptibility artifacts. These artifacts induce distortions which would otherwise prohibit accurate registration of diffusion-weighted and T1 images, and therefore complicate alignment with cortical segmentation. Combining the two polarity-reversed scans allows researchers to estimate an off-resonance field which is then applied to produce a corrected image.

### 2.2. Data processing

The first stage of our analysis consisted of segmentation with Freesurfer and NODDI parameter fitting [12] partially carried out with the pipeline described in [26]. Specifically, we denoise diffusion-weighted MRI data with a non-local means filter and fit NODDI parameters orientation dispersion index (ODI), isotropic volume fraction $\left(f_{\text{iso}}\right)$ and fractional intracellular volume $\left(f_{\text{icvf}}\right)$, and fiber orientation, using a non-linear method accelerated via the spherical mean technique [27]. Since we specialize to gray-matter regions, we fit with a parallel diffusivity of 1.1×10^−3^ mm^2^ s^−1^, which optimizes gray-matter fitting. Microstructurally, ODI quantifies the variation of neurites’ angular orientation in the voxel, $f_{\text{iso}}$ the fraction of the signal attributed to CSF, and $f_{\text{icvf}}$ the fraction attributed to the intracellular compartment, i.e., the space bounded by the neurite membranes. In this work, we utilize the the Desikan-Killiany [28] cortical atlas, which is a 34-region lateralized cortical parcellation. We acquire atlases for each subject from T1 images with Freesurfer v. 5.3.0.

Behavioral measures used in this study were sourced from the HCP-YA 1200 subject release behavioral dataset [22]. In this study, we specialize to NIH Toolbox tests under the “Cognition” category. These scores comprise oral reading (ReadEng), picture vocabulary (PicVocab), picture sequence (PicSeq), dimensional change card sorting (CardSort), pattern comparison processing speed (ProcSpeed), “flanker” (Flanker) and list sorting (ListSort) tests [29, 30]. The oral reading test quantifies participants’ abilities to accurately pronounce words and letters; the picture vocabulary test quantifies participants’ vocabulary and general knowledge abilities by presenting subjects with a recording of a word and asking them to select one of four photos which most closely matches the word pronounced; the picture sequence task is an episodic memory test involving the recall of sequences of illustrations of objects and activities; the dimensional change card sort task is a cognitive fluidity measure comprised of sorting cards according to one property or dimension (e.g., shape), and subsequently sorting along another dimension (e.g., color); the “flanker” task measures inhibitory control and attention abilities by asking participants to concentrate on one stimulus whilst ignoring flanking stimuli. The pattern comparison processing speed task measures how quickly the subject can determine whether two side-by-side images are identical; lastly, the list sorting task is a measure of working memory involving recalling and sorting objects presented either orally or visually according to a given criterion, e.g., size. Each of these measures is normalized to national averages, such that a score of 100 denotes the national average, and one standard deviation is scaled to 15 points. Age-adjusted scores are derived by computing national averages for each age band; age bands are separated by year for ages 3–17, and into eight bands (18–29, 30–39, 40–49, 50–59, 60–69, 70–79, 80–85) for adults. We utilize age-adjusted scores to control for the correlation between age and NODDI diffusion measures, as is demonstrated for hippocampal regions in [7].

### 2.3. PCA analysis

**Figure 2:**
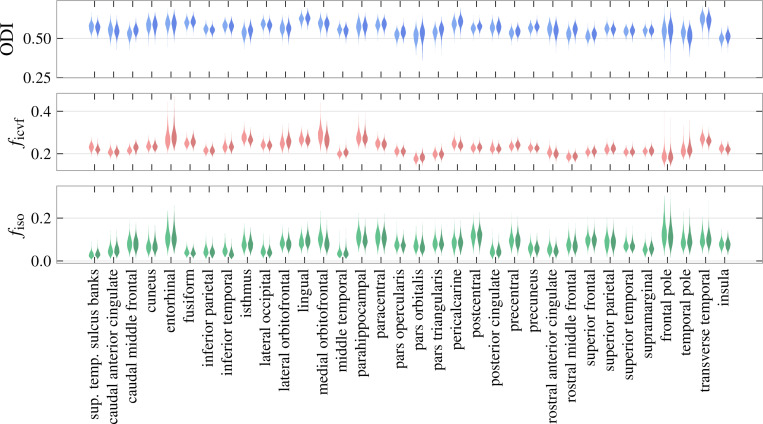
Distribution of mean per-region microstrucutural values across subjects. Extremal values corresponding to misregistration or distortion artifacts have been filtered. This is an intermediate result; principal component analysis is performed on these distributions to produce the general factors with weights pictured in Fig. 3.

Our aim is to extend the method of [21] to gray-matter imaging by introducing a general gray-matter microstructure factor (GMF), constructed by conducting a PCA analysis of individual NODDI parameter estimates across gray-matter regions. More specifically, each GMF is derived from region-averaged NODDI parameters $f_{\text{iso}}$, $f_{\text{icvf}}$ and ODI. PCA is performed along the dimension indexing cortical region, and the first principal component is selected as the general factor. See Fig. 2 for the aggregated per-region NODDI estimates for all subjects. This generates, for each subject, three values corresponding to a weighted average of each NODDI parameter. We remind the reader that each subject’s GMF is thus guaranteed to lie in the direction corresponding to maximal variance among subjects within the space of cortical regions. The role of this microstructure factor in driving cognitive effects is suggested by significant correlations with a subset of age-adjusted cognitive scores (obtained from HCP-YA data release). We utilize age-adjusted scores in our study so as to eliminate age as an additional covariate.

We additionally filter out voxels with extremal values of ODI and $f_{\text{icvf}}$ (below 0.03 and above 0.95). These values are anatomically unreasonable but can appear due to misregistration, typically in inferior regions such as the entorhinal cortex and temporal pole due to distortion from the nasal cavity. Alternatively, high values can appear in single-voxel loci as artifacts from the NODDI fitting scheme, i.e., local minima in the optimization procedure. The thresholds were chosen by visually inspecting the distributions in microstructure estimates and approximating the locations of minima that signal bimodality.

One source of confounding when regressing against parameters derived from finite brain regions is the partial volume effect, or partial voluming. This is expected to be most pronounced for isotropic volume fraction $f_{\text{iso}}$ but may also confound other NODDI parameter estimates (see Table 2). Indeed, previous work has revealed that DTI parameters, for instance, are susceptible to partial voluming, and it is suggested to control for region volume [31]. In the present work we achieve this by including a ‘weighted-average volume’ (WAV) covariate for each subject. The weighted-average volume is computed as follows. For a given subject, we compute the volume of each region in voxels. Then, we sum over these volumes, weighing each regions by the corresponding coefficient of the first PCA component, i.e., the microstructure factor. Lastly, we normalize by the number of regions. More precisely, let ${\text{w}}^{\left(1\right)}=\left(w_{1}^{\left(1\right)},w_{2}^{\left(1\right)},\cdots ,w_{N}^{\left(1\right)}\right)$ for N cortical regions, in our case 68, be the weights of the first PCA component. Then the weighted average volume is

(1) $${\tilde{v}}_{s}=\frac{1}{\sum_{i}w_{i}^{\left(1\right)}}\sum_{i}w_{i}^{\left(1\right)}v_{s,i}$$

where $v_{s,i}$ is the volume of region i for subject s. We emphasize that this definition implies that the weighted average volume depends on PCA component and thus on the particular microstructural parameter in question.

### 2.4. Statistical analysis

**Figure 3:**
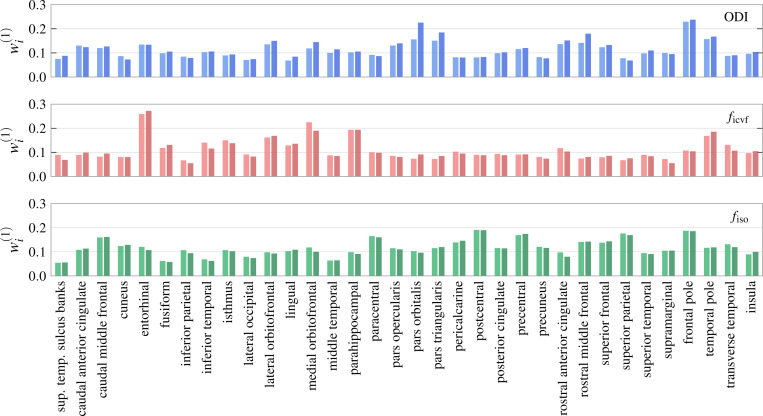
General factor weights corresponding to the first principal components of the distributions pictured in Fig. 2. The height of each bar corresponds to the entry of eigenvector associated with that region, i.e., how much variation in microstructure for that region contributes to global variation.

**Figure 4:**
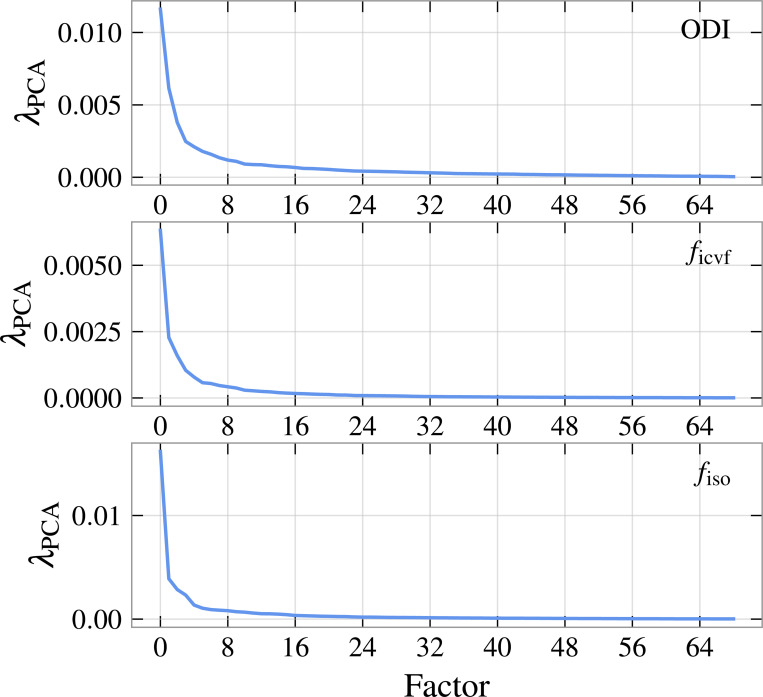
Scree plots for the PCA analysis of the mean microstructural parameters for each region, Fig. 2.

We perform PCA on each of the three scalar NODDI parameters, orientation dispersion index (ODI), CSF compartment fraction $\left(f_{\text{icvf}}\right)$, and neurite compartment fraction $\left(f_{\text{iso}}\right)$. Scree plots displaying the eigenvalues of the correlation matrix are displayed in Fig. 4. As can be observed from the plot, the first few ODI factors explain significant variation in the data; indeed we find that the first factor explains only 22.6% of the variance, suggesting a general ODI factor is less well defined than the other microstructure parameters. Specifically, $f_{\text{icvf}}$ and $f_{\text{iso}}$ have explained variances of 34.6% and 40.4% respectively, meaning that a single-factor solution is particularly strong for CSF compartment fraction. Factor weights are displayed in Fig. 3. We additionally report average between-region Pearson coefficients r in Table 1. We include a lateralized computation (r is not computed between regions in different hemispheres) to account for trends between analogous lateral regions.

We study the relationship between cognition and GMFs while controlling for the partial volume effect by performing multiple linear regression with GMF as response variable and cognitive score and weighted average volume (WAV) as predictors. We standardize the data so that all measures are in units of the standard deviation, and study correlations between each GMF and picture vocabulary, oral reading, list and card sorting, flanker, picture sequence, and processing speed scores. We perform a multiple-comparisons correction on the 21 *p*-values produced by these statistical tests. As the cognitive scores are positively correlated, we opt to use Benjamini-Hochberg false discovery rate (FDR) correction, which assumes the statistical tests are either independent or positively correlated [32, 33]. We note that the weighted-average volume covariate is not FDR-corrected.

We study the structure of the significant trends observed by performing the following posthoc tests targeting trends across cortical regions. Firstly, we study the trends between cognitive scores significantly correlated with the GMFs and mean microstructure estimates for each cortical region. We additionally investigate the presence of trends that fail to be captured by the GMFs by, for each significant cognitive score and microstructure parameter, performing multiple regression over all regions with the inclusion of the GMF as a covariate. Results are reported in the following section.

## 3. Results and Discussion

As described in the previous section, we extract gray-matter general microstructure factors (GMFs) for isotropic volume fraction $f_{\text{iso}}$, $f_{\text{icvf}}$, and ODI, and inspect trends between these factors and cognitive scores collected from the NIH Toolbox, controlling for partial-voluming by including a weighted-average volume (WAV) covariate defined in Sec. 2.3. We find a statistically significant relationship between $f_{\text{iso}}$ and card sorting (CardSort), oral reading (ReadEng), and picture vocabulary (PicVocab) scores. Correlation matrices for these three pairs are displayed in Table 2. We observe that the correlations between $f_{\text{iso}}$ and CardSort are weaker than the correlations between $f_{\text{iso}}$ and ReadEng and PicVocab. We additionally observe correlations between weighted-average volume and CardSort of comparable magnitude to the correlation between $f_{\text{iso}}$ and CardSort. Some correlation is observed between weighted-average volume and ReadEng and PicVocab, but smaller than correlations between GMF and cognitive score. The presence of correlations between cognition and weighted-average volume motivates the inclusion of volume as a covariate. FDR-corrected *p*-values and r coefficients are displayed in Table 3. Correlations between each of the GMFs and all other parameters are nonsignificant.

We investigate the detailed structure of the correlation between the cognitive scores and GMFs; in particular, we compute the correlations between microstructure estimate means for individual regions and the significant cognitive scores. We find that for $f_{\text{iso}}$, 54 regions are significantly correlated with ReadEng (4.64e−8 < *p* < 0.048) with trends comparable to the GMF (0.061 < *r* < 0.167), and 59 regions are significantly correlated with PicVocab (4.97e−8 < *p* < 0.0367), likewise with trends comparable to the factor (0.166 < *r* < 0.064). We note PicVocab and ReadEng share 51 significant regions in common. On the other hand, $f_{\text{iso}}$ has 26 regions significantly correlated with CardSort, with 0.001 < *p* < 0.047 and 0.061 < *r* < 0.100. These results confirm that there is a significant trend in which many regions participate. To test how much of this trend is conveyed by the GMF we control for the general factor and study the correlation between cognitive score and region estimate. We expect these correlations to reveal any trends not captured by the factor. In this case $f_{\text{iso}}$ correlates significantly with CardSort for only 4 regions, with only 2 of those regions trending in the same direction as the factor. Similarly, only 4 regions correlate $f_{\text{fiso}}$ significantly with ReadEng, and only 2 correlate significantly with PicVocab.

We conclude that there is a significant structure-function relationship between global variation in cortical isotropic volume fraction $f_{\text{iso}}$ and performance in oral reading and picture vocabulary tests. A significant trend is also observed for the general $f_{\text{iso}}$ factor and card sorting test performance, although this is carried by correlation with a smaller group of regions, indicating that the trend is less adequately captured by a global measure of tissue microstructure. This is reflected in the larger *p*-value for CardSort reported in Table 3. We expect these results are robust to partial-voluming thanks to the inclusion of weighted-average volume as a covariate. The observed trend relating $f_{\text{iso}}$ to cognition is expected as previous work has established, for example, relation between $f_{\text{iso}}$ and cognitive impairment [34], or decreased $f_{\text{iso}}$ in language-processing regions for subjects with language impairments [35].

We close this section by listing some open questions raised by our analysis. In this study we specialized to the NODDI microstructure model; however, several other models capable of capturing gray-matter microstructure are also available. This includes elementary signal representations such as diffusion tensor imaging (DTI), or, more relevant to the present case, more sophisticated models targeting gray matter, such as Soma and Density Neurite Imaging (SANDI) [11]. One could carry out a multimodal study systematically comparing general factors derived from each model; this could shed light on the sensitivity of each model to global variations, as well as reveal whether such global variations in sophisticated models capture structure-function trends differently. We note that, for richer models such as SANDI, higher *b*-value acquisitions are required, and the dataset used in this work would be insufficient.

We also mention that our analysis is constrained by the properties of PCA dimensionality reduction. While such linear approximations extract simple and interpretable trends, PCA assumes that the direction of maximal variation is a sound probe of anatomical signal and that inter-region trends are linearly correlated and orthogonal. Contrast with the case where, for example, the direction of maximal variation is due to noise. It remains an open question whether better results might derive from more sophisticated dimensionality reduction techniques such as independent component analysis (ICA).

Further, we encountered many subjects with small misregistrations that introduced artifacts to the dataset, typically originating in inferior regions. The method used in this work for omitting misregistered voxels was crude—we filter out voxels with microstructure estimates below and above a certain threshold, motivated by anatomical constraints. On the other hand, identifying the affected voxels by hand quickly becomes impractical in studies with a large number of subjects, such as ours. Thus, there is demand for a more refined and efficient technique for flagging misregistered regions.

Lastly, we note that the technique explored in this study is amenable to several variations. While we generated general factors from trends across regions defined by the Desikan-Killiany atlas, one might compare against general factors derived from other segmentations. Alternatively, one may sample microstructure parameters from ROIs defined surface maps, or eschew an ROI approach and sample voxels or surface map vertices directly. In the latter case, the dimensionality of the PCA problem can increase prohibitively; one may reduce the dimensionality by averaging microstructure parameters over subdivisions of a defined size, for example, to spheres of a chosen radius. By inspecting the results of PCA analysis as the subdivision size is tuned, one may in principle study the dependence of trends in microstructural parameters to anatomical length scale.

## 4. Conclusion

In this work, we demonstrated that gray-matter general microstructure factors derived from NODDI parameters capture meaningful variation in cortical tissue organization and are significantly associated with specific cognitive domains. Using a large cohort from the Human Connectome Project, we showed that global $f_{\text{iso}}$ factors constructed via PCA explain a substantial portion of inter-individual variability. These factors were robustly linked to performance in sorting, reading, and vocabulary tasks, with the $f_{\text{iso}}$ factor exhibiting broad cortical associations with oral reading and vocabulary tasks, and more localized associations with card sorting tasks. Together, these findings highlight the utility of general microstructure factors as global indicators of cortical organization and suggest that they may provide a complementary framework for studying structure–function relationships beyond region-specific analyses.

## Figures and Tables

**Figure 1:**
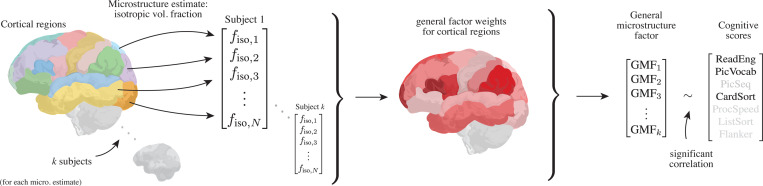
We study the relationship between global trends in NODDI gray-matter microstructure estimates and cognition using the procedure outlined by the figure. For each subject, we construct a vector with entries equal to per-region mean microstructure estimates. This produces, for each subject, a vector with one entry per cortical region. We then use PCA analysis to compute a general microstructure factor (GMF) which assigns weights to each cortical region corresponding to that region’s participation in global variation in microstructure parameter. Each subject is thus assigned a GMF value. We then study trends between GMF and cognition using NIH Toolbox cognitive scores, and find a significant correlation between GMF and three cognitive scores.

**Figure 5:**
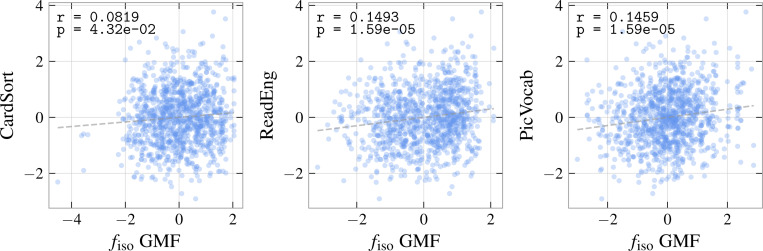
Lines of best fit for each of the significant trends observed. The dependent axis plots the general microstructure factor (GMF) of the corresponding NODDI parameter.

**Table 1:** Mean inter-region Pearson coefficients for each microstructural parameter. We include a lateralized mean Pearson coefficient where r is not computed for regions between hemispheres, as analogous lateral regions are observed from Fig. 2 to be highly correlated. This may be interpreted as a quantification of the global correlation that microstructural parameters experience throughout the cortex.

	ODI	$f_{\text{icvf}}$	$f_{\text{iso}}$
Mean inter-region r	0.232	0.382	0.418
Mean inter-region r (lat.)	0.242	0.385	0.423
Explained variance	0.226	0.346	0.404

**Table 2:** Correlation matrices for general microstructure factor (GMF), cognitive score, and weighted-average volume. We observe comparable correlations between volume and card sorting score, inspiring us to correct for volume effects by residualizing.

	$f_{\text{iso}}$	Factor	CardSort	WAV
$f_{\text{iso}}$ Factor		1.000	0.082	−0.036
CardSort		0.082	1.000	0.060
WAV		−0.036	0.060	1.000

	$f_{\text{iso}}$	Factor	ReadEng	WAV
$f_{\text{iso}}$ Factor		1.000	0.149	−0.036
ReadEng		0.149	1.000	0.033
WAV		−0.036	0.033	1.000

	$f_{\text{iso}}$	Factor	PicVocab	WAV
$f_{\text{iso}}$ Factor		1.000	0.146	−0.036
PicVocab		0.146	1.000	0.035
WAV		−0.036	0.035	1.000

**Table 3:** Results of multiple regression of microstructure factor against cognitive score and weighted-average volume.

		p	r
$f_{\text{iso}}$	CardSort	0.043	0.084
	WAV	0.184	−0.041
$f_{\text{iso}}$	ReadEng	1.594e−5	0.150
	WAV	0.18	−0.041
$f_{\text{iso}}$	PicVocab	1.594e−5	0.147
	WAV	0.178	−0.041
